# Supplementary material for: Association between oral microbiome and seven types of cancers in East Asian population: a two-sample Mendelian randomization analysis
Source: Front Mol Biosci. 2023 Nov 21;10:1327893. doi: 10.3389/fmolb.2023.1327893 (PMC10702768; doi:10.3389/fmolb.2023.1327893)
Supplement: Supplementary file 1 [file DataSheet1.ZIP › Supplementary Materials/MR plots for tongue/tongue═╝/Ovarian cancer/pheno.1803_to_ovarian cancer_forest.pdf]

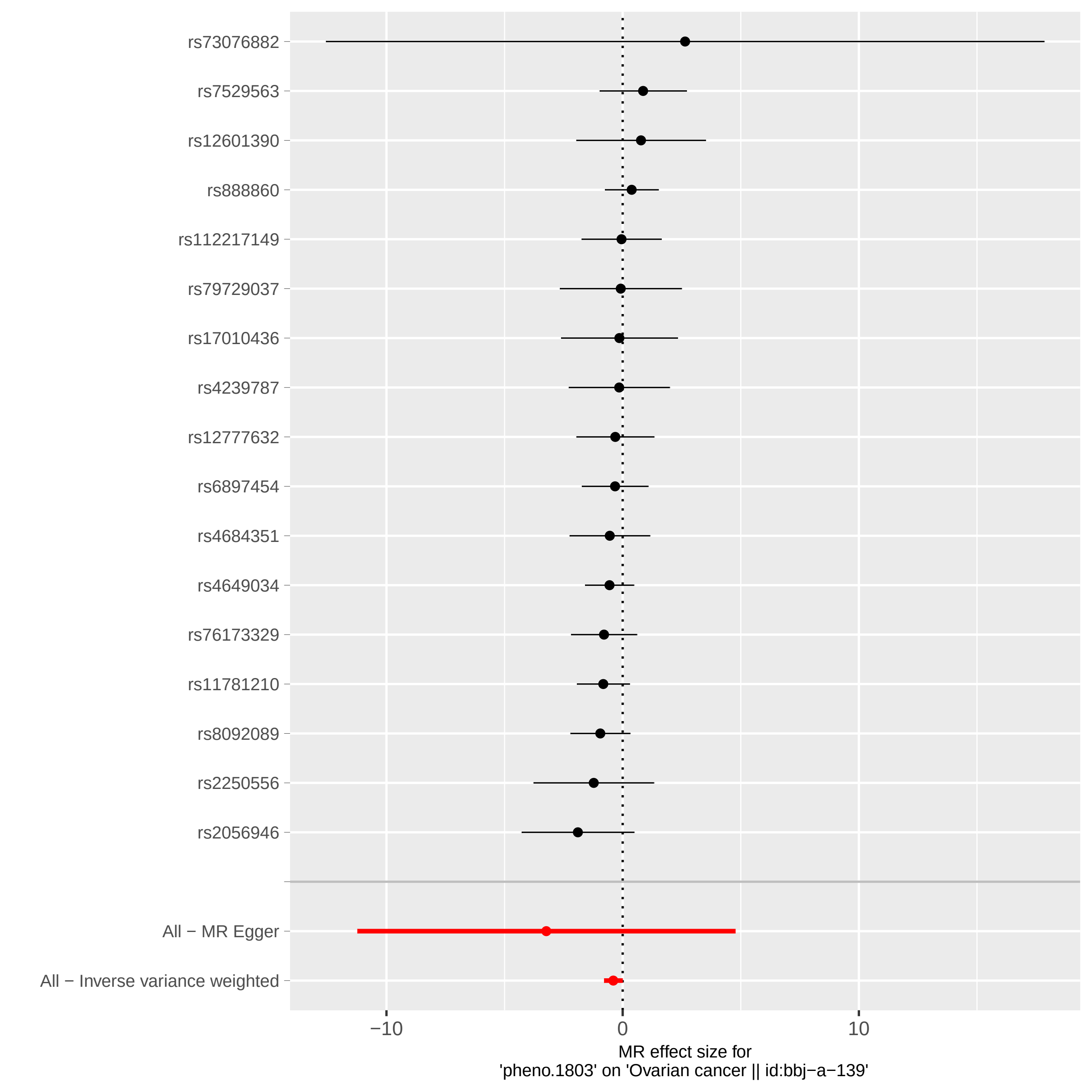
rs73076882
rs7529563
rs12601390
rs888860
rs112217149
rs79729037
rs17010436
rs4239787
rs12777632
rs6897454
rs4684351
rs4649034
rs76173329
rs11781210
rs8092089
rs2250556
rs2056946
All − MR Egger
All − Inverse variance weighted
−10
0
10
MR effect size for
'pheno.1803' on 'Ovarian cancer || id:bbj−a−139'
